# MS-CLSTM: Myoelectric Manipulator Gesture Recognition Based on Multi-Scale Feature Fusion CNN-LSTM Network

**DOI:** 10.3390/biomimetics9120784

**Published:** 2024-12-23

**Authors:** Ziyi Wang, Wenjing Huang, Zikang Qi, Shuolei Yin

**Affiliations:** 1 School of Materials Science and Engineering, Central South University of Forestry and Technology, Changsha 410004, China; 20221200241@csuft.edu.cn (Z.W.); 20231100273@csuft.edu.cn (Z.Q.); 20231200266@csuft.edu.cn (S.Y.); 2 School of Mechanical and Intelligent Manufacturing, Central South University of Forestry and Technology, Changsha 410004, China

**Keywords:** sEMG gesture recognition, deep learning, multi-scale feature fusion, real-time prediction

## Abstract

Surface electromyography (sEMG) signals reflect the local electrical activity of muscle fibers and the synergistic action of the overall muscle group, making them useful for gesture control of myoelectric manipulators. In recent years, deep learning methods have increasingly been applied to sEMG gesture recognition due to their powerful automatic feature extraction capabilities. sEMG signals contain rich local details and global patterns, but single-scale convolutional networks are limited in their ability to capture both comprehensively, which restricts model performance. This paper proposes a deep learning model based on multi-scale feature fusion—MS-CLSTM (MS Block-ResCBAM-Bi-LSTM). The MS Block extracts local details, global patterns, and inter-channel correlations in sEMG signals using convolutional kernels of different scales. The ResCBAM, which integrates CBAM and Simple-ResNet, enhances attention to key gesture information while alleviating overfitting issues common in small-sample datasets. Experimental results demonstrate that the MS-CLSTM model achieves recognition accuracies of 86.66% and 83.27% on the Ninapro DB2 and DB4 datasets, respectively, and the accuracy can reach 89% in real-time myoelectric manipulator gesture prediction experiments. The proposed model exhibits superior performance in sEMG gesture recognition tasks, offering an effective solution for applications in prosthetic hand control, robotic control, and other human–computer interaction fields.

## 1. Introduction

Surface electromyography (sEMG) signals are the superposition of all motor unit action potential (MUAPT) sequences in the electrode coverage area during human muscle activity, and this bioelectrical signal has the role of predicting the human movement intention [1], which has been widely used in non-invasive human–computer interaction systems such as robotic hand control, wheelchair control, virtual interaction, and disability rehabilitation robots [2,3,4]. sEMG-based myoelectric manipulators use methods such as machine learning to decode the sEMG generated by gesture movements and make predictive judgments about the movement patterns, thus realizing human–computer interaction [5,6,7,8], so the sEMG-based myoelectric manipulator gesture recognition problem belongs to the multi-classification problem in pattern recognition, and there are roughly two approaches, i.e., based on the traditional machine learning method and based on the deep learning methods. Traditional machine learning methods usually extract time-domain, frequency-domain, or time–frequency-domain features from sEMG, and after the features are downscaled, gesture recognition is carried out by traditional classification methods, such as support vector machine (SVM) [9,10,11,12], the fuzzy method (FZ), and the linear discriminative method (LDA) [13,14,15]. These methods have achieved good results on the task of recognizing a small number of gestures, but the classification accuracy decreases significantly with the increase in gesture categories.

Deep learning methods have powerful non-linear expressive ability and self-learning ability, which can realize our automation requirements for complex transaction processing, and currently have made revolutionary breakthroughs in image recognition, speech recognition, etc. The recognition effect is far greater than that of the previous related technologies. In recent years, deep networks have been gradually used for decoding the grasping gestures of myoelectric dummy hands based on sEMG signals [16,17,18]. Deep-learning-based sEMG gesture recognition methods improve classification efficiency by automatically extracting deep features from sEMG through neural networks. Geng et al. [19] constructed a ConvNet architecture that takes instantaneous surface EMG as input, achieving a gesture recognition accuracy of 77.8% on the Ninapro DB2 dataset and verifying the feasibility of using sEMG for gesture recognition. Wei et al. [20] proposed a staged recognition method, where sEMG signals are first decomposed into smaller segments (streams), and features are learned independently by CNNs. The features are then fused to complete gesture recognition, achieving an accuracy of 81.7% on the Ninapro DB1 dataset. Additionally, Wei et al. [21] combined traditional feature sets with deep learning models to construct a multi-view representation of sEMG signals and modeled it with parallel CNNs, improving accuracy to 83.7%. These methods primarily focus on extracting the spatial features of EMG signals but overlook their crucial temporal characteristics, which limits model performance. To fully exploit the temporal features of sEMG, hybrid architectures have gained popularity. For example, Wu et al. [22] proposed a combined LSTM-CNN architecture, achieving 75.16% accuracy on the Ninapro DB5 dataset, demonstrating the feasibility of integrating temporal modeling with convolutional feature extraction. Kim et al. [23] introduced a CNN-LSTM architecture that converts sEMG into two-dimensional spectrograms for input, achieving an accuracy of 83.91%. However, this conversion process leads to some loss of the original time-domain information. Hu et al. [24] proposed a hybrid CNN-RNN architecture with an attention mechanism, achieving an average accuracy of 84.80% on the Ninapro DB1 dataset. Wang et al. [25] improved temporal feature extraction efficiency by combining temporal depthwise convolutional with Transformer (TDCT). Zhang et al. [26] integrated a convolutional vision Transformer with a stacked ensemble learning approach, significantly enhancing model robustness in complex gesture recognition tasks. However, sEMG data typically consist of small sample sizes and sparse signals, which can lead to model overfitting. Hybrid architectures and Transformer models often have complex structures, large parameter counts, and high computational demands, limiting their applicability to small datasets.

sEMG signals reflect the synergistic effects of local electrical activity from muscle fibers and overall muscle groups, containing detailed local information and broader global patterns. Some CNN models use single-scale convolutional kernels to learn features from sEMG. For example, Zhan et al. [27] achieved gesture recognition of sEMG signals using a single-scale convolutional kernel, demonstrating CNNs’ superior performance in capturing local features. Similarly, Lee et al. [28] used artificial neural networks (ANNs) to classify sEMG, achieving high accuracy. In gesture recognition, some gestures may trigger signal changes in localized muscle regions, while others involve larger muscle groups. The fixed-scale feature extraction approach of ANNs and single-scale CNNs struggles to comprehensively capture local details and global patterns, particularly in tasks requiring simultaneous attention to subtle variations and long-term dependencies. Multi-scale convolutional methods integrate convolutions across different receptive fields to better capture both local and global features, enhancing the ability to differentiate between gestures. These methods are increasingly applied to sEMG processing tasks. For instance, Han et al. [29] proposed the MKFF-CNN model, which combines multi-scale convolutional kernels with an early–late fusion strategy. This model achieved an accuracy of 97.65% on the g-Force dataset, marking a 6.54% improvement over single-scale convolutional networks. Zhang et al. [18] proposed a multi-scale multi-branch CNN algorithm that effectively captures multi-level features in sEMG by combining a multi-branch structure with multi-scale convolutional kernels, achieving 85.2% accuracy on the Ninapro DB5 dataset. Luo et al. [30] integrated a multi-scale attention mechanism with ResNet50 and proposed the InRes-ACNet model, which optimizes performance on sparse signals. Fratti et al. [31] combined transfer learning with multi-scale CNNs to develop the MSCNN model, enhancing cross-user adaptation and demonstrating the effectiveness of multi-scale feature fusion in capturing both local details and global patterns. The multi-scale convolutional approach enhances adaptability and accuracy in gesture recognition tasks, offering an effective solution for processing myoelectric signals.

This paper proposes an MS-CLSTM (MS Block-ResCBAM-Bi-LSTM) network model based on multi-scale feature fusion. The network consists of two parallel branches, each containing a multi-scale feature extraction module (MS Block) and a residual convolutional block attention mechanism (ResCBAM). The bidirectional long short-term memory (Bi-LSTM) network models the temporal nature of the features fused from the two branches and captures the temporal characteristics of sEMG signals. The MS Block extracts large-scale and small-scale features of the sEMG signal using parallel convolutional kernels of different sizes. The large-scale features capture the contextual information of sEMG signal variations and reflect the correlations between multiple channels, while the small-scale features focus on the local details of the sEMG and the correlations between neighboring channels. ResCBAM, which integrates CBAM with Simple-ResNet, further enhances the multi-scale features of the sEMG signals, increasing the diversity of the information contained in the features. CBAM allows the network to pay more targeted attention to the salient features of different gestures. Meanwhile, Simple-ResNet reduces the number of network layers and parameters, helping to mitigate the overfitting issues common in traditional deep networks when dealing with small samples and sparse data. By combining these modules, the MS-CLSTM network model enables real-time control of myoelectric manipulators, demonstrating potential advantages in sEMG signal processing and similar fields.

The rest of the paper is organized as follows: Section 2 describes the two datasets used in this paper and the signal preprocessing. Section 3 constructs the MS-CLSTM model. Section 4 experimentally validates the effectiveness of MS-CLSTM for multi-scale feature fusion networks. Section 5 summarizes the contents of this paper.

## 2. Methods

**Figure 1:**
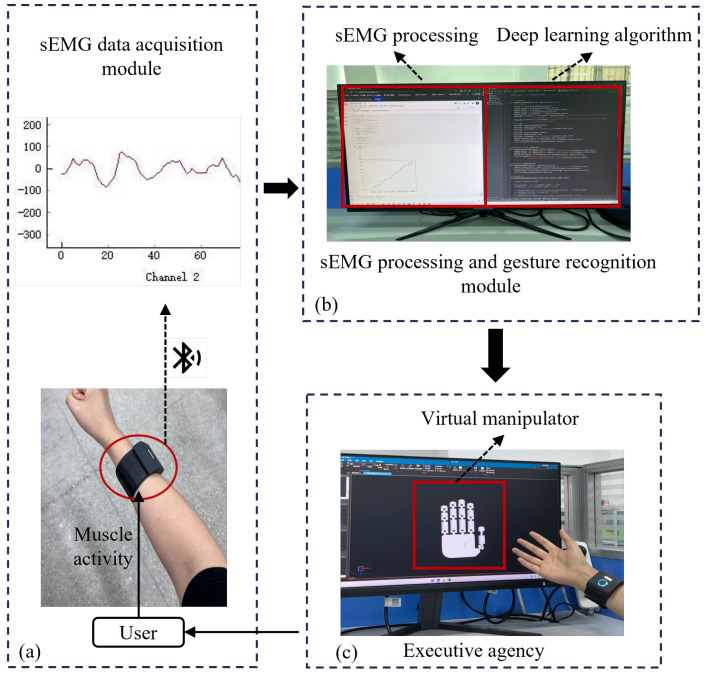
Real-time control system for myoelectric manipulators based on sEMG. (**a**) sEMG data acquisition module; (**b**) sEMG processing and gesture recognition module; (**c**) Executive agency (Virtual manipulator).

The real-time control system of the manipulator based on sEMG is shown in Figure 1. The system includes three main modules: signal acquisition module, signal processing and gesture recognition module, and actuator. The signal acquisition module collects the sEMG of the arm in real time through a wireless 8-channel sEMG armband, and the signals are then transmitted to the computer in real time by Bluetooth. The computer communicates with the Bluetooth serial port through specific software and acquires the current sEMG data based on the serial communication protocol. The signal processing and gesture recognition module performs preprocessing such as denoising and filtering the acquired signals, followed by parsing the user’s gesture categories using deep learning algorithms. After the gesture decoding is completed, the system generates the corresponding control commands according to the recognized gesture categories and directly drives the robot to complete the specified actions.

### 2.1. Air-Band sEMG Data Acquisition

**Figure 2:**
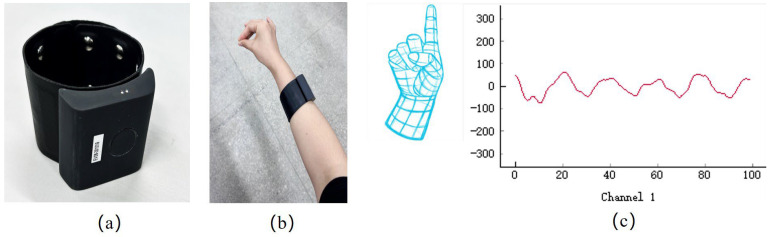
sEMG acquisition: (**a**) Air-Band myoelectric bracelet; (**b**) Gesture action with Air-Band myoelectric bracelet; (**c**) sEMG acquisition.

The sEMG acquisition device is the Air-Band sEMG bracelet from Zhejiang Zoeling Technology Co. (Tianren Building, 25th Floor, Room 2503, Xiaoshan District, Hangzhou, Zhejiang Province, China). The Air-Band sEMG bracelet adopts a strap type and one-piece modular design, with a strap length of about 19 cm and a width of about 3.5 cm, and is made of leather material, which makes it comfortable to wear, as shown in Figure 2a,b. The bracelet uses metal electrodes, configured with 3 groups of 8 electrodes in total, with 8 channels and a sampling rate of 1000 Hz. It is waterproof and provides a built-in vibration feedback function. At the same time, the bracelet has a built-in high-precision IMU sensor for motion capture. The device can last more than 5 h in normal operation and 7 days in standby (hibernation) and supports magnetic contact charging. The device is equipped with an acquisition data visualization interface, as shown in Figure 2c, which includes the current acquisition round, the current acquisition gesture information, and the real-time data of eight channels.

**Figure 3:**
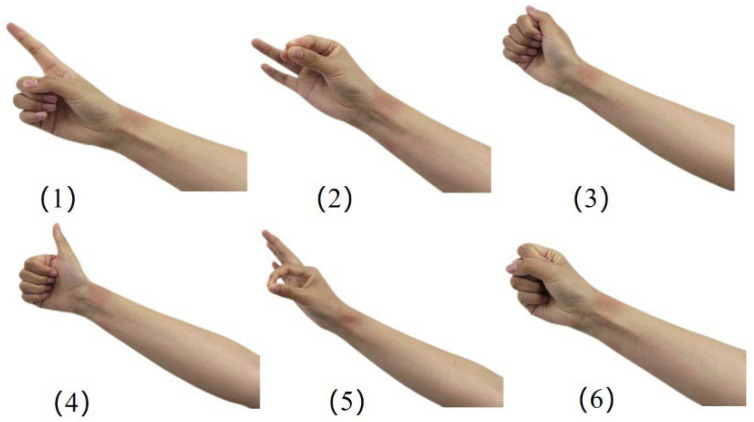
Six hand movements: (**1**) index finger movement, (**2**) three-finger grip moving up and down, (**3**) left and right slide of the thumb, (**4**) thumbs up, (**5**) two-finger grasping and releasing, (**6**) clenched fist.

Six gestures were captured for each subject, which were (1) index finger movement: can be used to click to select an object or option, (2) three-finger grip up and down movement: can be used to zoom in and out of the selected object (object or background), (3) left and right slide of the thumb: can be used for forward and backward adjustments of the steps, (4) vertical thumb: can be used to return or exit the operation, (5) two-finger grasp release: can be used for grasping the objects and release, (6) clenched fist: can be used to select a specific pop-up window, and the specific gestures are shown in Figure 3.

### 2.2. sEMG Data Processing

sEMG is a non-linear non-stationary time series signal generated by weak action potentials generated by muscle fibers on the surface of the skin during skeletal muscle contraction, which can reflect information related to muscle and body behavior. Like the measurement of other physiological electrical signals, the sEMG is easily corrupted by noise, with three types of noise, namely, industrial frequency interference, Gaussian white noise, and baseline drift, which leads to difficulties in signal analysis and low signal-to-noise ratio. To better analyze the sEMG, it needs to be preprocessed. The preprocessing process mainly includes filtering and Z-Score normalization. The amplitude of the sEMG collected by the electrode sensors is usually between 15 and 100 μV, and the energy of the useful signals is mainly distributed between 10 Hz and 500 Hz, so this paper adopts a fourth-order Butterworth filter for the band-pass filtering, and the passband boundary is 10–500 Hz.

The data value of the filtered sEMG is extremely small, and the difference between the data is usually 100 times, which will directly affect the experimental results, so the Z-Score normalization is used to convert the sEMG to a fixed range, and its mathematical formula is shown in Equation (1):

(1) $$z=\frac{x-\mu }{\sigma }$$

where x is the original value of sEMG, z is the standardized value after conversion, μ is the mean value of the overall sample in the sEMG, and σ is the standard deviation of the overall sample in the sEMG. Considering the differences in acquisition locations and the physiological properties of muscle tissue, the sEMG data between different channels are somewhat different, so the sEMG data of each channel are individually normalized. Z-Score normalization normalizes the values of the true signals from around 10^−4^ to [0, 1], reduces the influence of extreme values in the signals acquired by the same electrode, and increases the signal separability of the electrodes acquired by the electrodes with a low degree of stimulation.

### 2.3. Grayscale Diagram of sEMG

After the sEMG is preprocessed, it is decomposed into small window segments using a sliding window and a stepped window to obtain segmented sEMG images. Let the window length of the sliding window be w and the step window be s. The segmented sEMG image can be obtained after slicing, and the obtained image is recorded as $h\in R^{L\times T}$, where *L* is the amount of data recorded by a single channel, *T* is the number of acquisition channels, and the formula for calculating *L* is as follows:

(2) $$L=\frac{w\times f}{1000}$$

where w is the window length of the sliding window and f is the sampling frequency of the sEMG.

**Figure 4:**
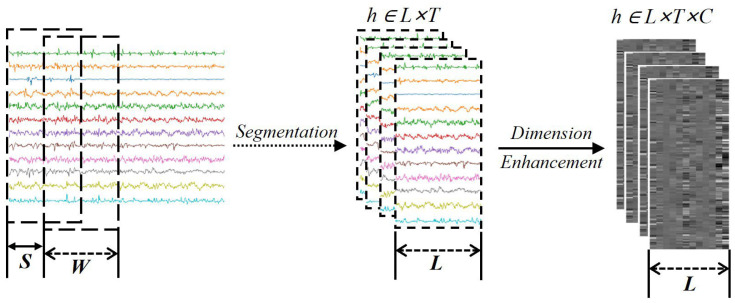
Grayscale diagram of sEMG. (Different colored lines in the figure represent data from 12 channels.)

In the subsequent feature extraction process, to avoid the feature information matrix between different channels being compressed into vectors and resulting in the loss of information, this paper uses two-dimensional convolution for feature extraction of sEMG, and two-dimensional convolution can effectively avoid the problem of loss of information by convolving along two directions in the feature extraction process. Taking the sEMG image as an example, the convolution along the L direction can obtain the features of single electrode channel signals, and the convolution along the T direction can obtain the spatial features of multiple channels. To comply with the two-dimensional convolution input, we carry out the dimension enhancement operation on the sEMG image, and the sEMG image obtained after dimension upgrading is $h\in R^{L\times T\times C}$, where L can be regarded as the length of the image, T can be regarded as the width of the image, C is the number of the feature channels of the image, and C=1 indicates that the image is a single feature channel, i.e., the image obtained after dimension upgrading is a single-feature-channel grayscale image, and the overall imaging process is shown in Figure 4.

## 3. Network Architecture

### 3.1. Overall Model (MS-CLSTM)

**Figure 5:**
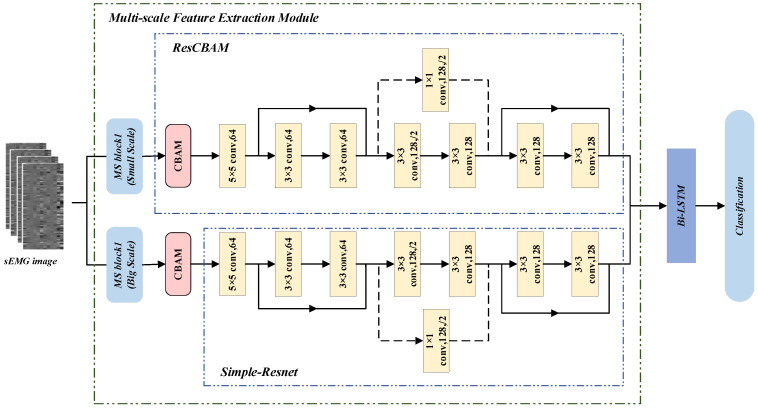
The overall framework of the MS-CLSTM.

The MS-CLSTM model based on multi-scale feature fusion proposed in this paper is an end-to-end model to adaptively extract multi-scale features from sEMG images for accurate classification. The overall network structure is shown in Figure 5. First, the sEMG image obtained after preprocessing is inputted into the multi-scale feature extraction network, which is a parallel structure and consists of the multi-scale feature extraction module (MS Block) and ResCBAM. The two parallel branches are divided into the Small Scale branch and Big Scale branch according to the MS Block settings, the Small Scale branch extracts small-scale features of sEMG, and the Big Scale branch extracts large-scale features. ResCBAM is integrated by CBAM and Simple-ResNet, which is a part of ResCBAM that enables the network to pay more targeted attention to different gestures, focus on the salient features of different gestures, and help the model to understand and learn the feature representation of the input sEMG more deeply. Finally, the loss of feature information is mitigated by re-extracting temporal features from the fused features using Bi-LSTM.

### 3.2. Multi-Scale Block (MS Block)

The big-scale convolutional kernel can capture overall features, but it is not sensitive enough to capture detail features, while the small-scale convolutional kernel can capture detail features more efficiently but cannot capture overall features. Therefore, in this paper, we propose a Multi-Scale Block (Small Scale and Big Scale) to extract detailed and overall features of sEMG, respectively. The MS block is proposed based on the structure of Inception. Inception [32] is a classical feature extraction module, first proposed by GoogLeNet, which captures different features of sEMG by using convolution kernels with different sizes in parallel and pooling operations to capture feature information at different scales and splice them in the channel dimension, so that both local and global information of features can be considered at different scales, thus improving the model’s ability to learn from images.

**Figure 6:**
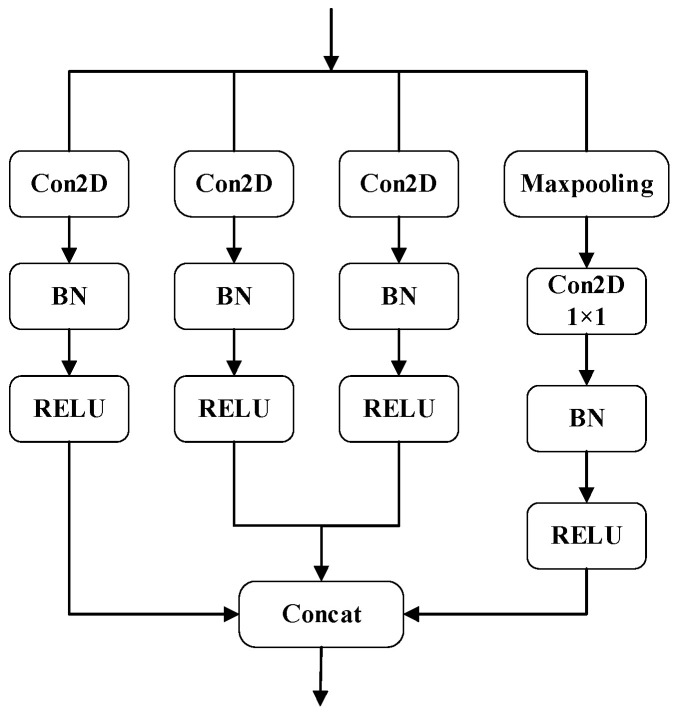
The structure of Multi-Scale Block.

The structure of the MS Block is shown in Figure 6. The first three branches are composed of convolutional kernels of different scales, where branch 1 and branch 2 are used to extract the overall and local information of the sEMG, and branch 3 is used to extract the correlation information between the channels. The convolutional kernel sizes of Small Scale and Big Scale are set up as shown in Table 1, and the convolutional kernels of 5 × 1 and 8 × 1 on the Small Scale are used to extract the detailed and locally changing features of sEMG within a short period. In Small Scale, 5 × 1 and 8 × 1 convolution kernels are used to extract the details of and local changes in sEMG in a short time range, and 1 × 2 convolution kernels are used to extract the correlation information between neighboring channels; in Big Scale, 10 × 1 and 20 × 1 convolution kernels are used to capture the global dynamics of the sEMG in a long time range as a result of the changes in the gestures, and 1 × 4 convolution kernels are used to extract the correlation information between multiple channels. Branch 4 then performs a maximum pooling operation to downsample the data to remove redundant information on the one hand and retain the important information of the feature maps on the other hand to improve the classification accuracy of the model. The length of the convolution kernel after the maximum pooling operation is 1 to maintain the same number of feature map channels as the first three branches. Finally, the features obtained from the feature extraction layer of the four branches are subjected to concatenation operation and the results are output to the subsequent modules.

The four-branch structure of the MS Block can effectively capture different scale feature information in the sEMG to better adapt to the complexity of sEMG images. In addition, the features extracted from each branch are merged in the channel dimension to form a rich and diverse feature representation, which provides more informative inputs for subsequent tasks.

### 3.3. Residual Convolutional Block Attention Mechanism (ResCBAM)

The Attention Model has become an important concept in neural networks in recent years. The Attention Model can mimic the mechanism of human visual attention, which has been widely used and deeply studied in different application areas [33,34]. The core idea is to enable neural networks to pay more attention to task-relevant information, thus improving the performance of the model. In this paper, ResCBAM is integrated by the Convolutional Block Attention Module (CBAM) and Simple-ResNet. The structure of ResCBAM is shown in Figure 5. Firstly, the CBAM is utilized to enhance the salient signal features, to make the processing of the data more focused on this part, and then Simple-ResNet is utilized further to enhance the deep representation of the model learning capability and mitigate the gradient vanishing problem through residual learning, making the network easier to train and converge.

**Figure 7:**
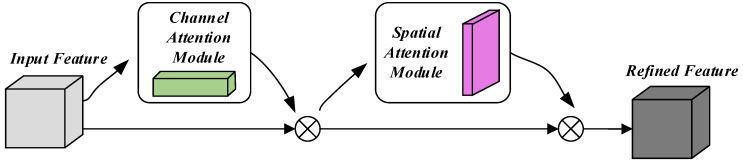
The network structure of the CBAM.

The overall structure of CBAM is shown in Figure 7, including two sub-modules, the Channel Attention Module and the Spatial Attention Module. The Channel Attention Module does not change the channel dimension, but compresses the spatial dimension, focusing on the meaningful information between the channels in the input image. The Spatial Attention Module does not change the spatial dimension, compresses the channel dimension, and focuses on the positional information of the target. Spatial attention is computed as follows:

(3) $$\begin{array}{c} M_{s}(F)=\sigma (f^{a\times a}[(AvgPool(F);MaxPool(F)]))\\ =\sigma (f^{a\times a}[F_{avg}^{s};F_{\max}^{s}])) \end{array}$$

where AvgPool represents the average pooling operation, MaxPool represents the maximum pooling operation, $M_{S}(F)\in {\mathbb{R}}^{H\times W}$ represents the spatial attention map, $F_{avg}^{S}\in {\mathbb{R}}^{H\times W\times 1}$ and $F_{\max}^{S}\in {\mathbb{R}}^{H\times W\times 1}$ represent the average and maximum pooled features in the feature channel, σ represents the sigmoid function, and $f_{a\times a}$ represents the convolution process of a convolution kernel with the size of a×a.

In CBAM, the spatial attention convolution kernel a×a is generally set to 7×7, while in this paper, considering the specificity of sEMG $h\in R^{L\times T\times C}$, the size of the convolution kernel a×a is set to 2×2, and the convolution kernel 2×2 is used to extract the features of the neighboring signals as well as the neighboring channels, and by adjusting the size of the convolution kernel a×a, it is ensured that the spatial attention part of the convolution operation is more in line with the spatial structure of the sEMG image, and it improves the model’s capability of effectively extracting the features of the sEMG image.

sEMG data belong to a small-sample dataset, and the sEMG exhibits sparse characteristics such as discontinuity and high volatility in the time and frequency domains, which is very likely to lead to overfitting of the model. To solve this problem, the Simple-ResNet structure is proposed in this paper, as shown in Figure 5. Compared with the original ResNet, Simple-ResNet has a shallower number of network layers, which allows the model to avoid overly complex feature extraction and prevents overfitting when dealing with small samples and sparse data. Meanwhile, Simple-ResNet retains the shortcut connection design of ResNet, which can directly transfer the input signal during the forward propagation of the model, enabling the model to better retain the information in the original sEMG and avoid feature loss in the sparse signal. Under the premise of guaranteeing the model performance, the structure enhances the adaptability to the sparsity and category imbalance problems of sEMG data.

### 3.4. Bidirectional LSTM (Bi-LSTM) for Temporal Feature Extraction

sEMG is highly temporal, so temporal networks can be used to extract the temporal features of the signals. Bi-LSTM [34] consists of two LSTM layers in opposite directions, which can simultaneously process the forward and reverse information of the input sequences along the time-domain direction. The outputs of each layer of the Bi-LSTM are not only dependent on the state of the hidden layer in the previous time point but also associated with the state of the hidden layer in the subsequent time point, thus fully capturing the temporal variations of the input signals. It is also associated with the hidden layer states at the subsequent time points, thus fully capturing the changes in the input signal in the time sequence. By integrating the information of the whole time series range, the model’s ability to perceive the gesture changes can be improved. In this paper, a shallow recurrent neural network is constructed with the help of Bi-LSTM, specifically, a layer of a bidirectional long short-term memory network is introduced, which can further model the temporal nature of the feature maps output from the multi-scale feature extraction network part, so that the classification results can make full use of the signal features on the temporal sequence. In the overall network structure, ResCBAM is designed to extract spatial features, while the introduced Bi-LSTM is responsible for the temporal modeling of these features.

Because the multi-scale feature extraction network at the front end of the backbone network obtains the features 1×128, the number of hidden layers of Bi-LSTM is set to 128. After the features are extracted by Bi-LSTM, the output of the sEMG feature maps is 1×256 and the features are deactivated by the dropout layer to alleviate the overall overfitting problem of the model.

## 4. Model Training and Experimental Results

In this paper, a total of two experiments were conducted: the first experiment was based on the publicly available datasets Ninapro DB2 and Ninapro DB4; the second experiment was based on robotic gesture recognition with an Air-Band myoelectric bracelet. Each experiment takes gesture recognition accuracy and overall classification accuracy as evaluation metrics, and the gesture recognition accuracy (Accuracy) of the subjects in the experiment is defined as:

(4) $$\mathrm{Accuracy}=\frac{\mathrm{Number}\;\mathrm{of}\;\mathrm{correct}\;\mathrm{classifications}}{\mathrm{Total}\;\mathrm{number}\;\mathrm{of}\;\mathrm{test}\;\mathrm{samples}}\times 100\%$$

The overall average recognition accuracy (Average accuracy) is defined as:

(5) $$\mathrm{Average}\;\mathrm{accuracy}=\frac{1}{N}\sum_{n=1}^{N}\mathrm{Accuracy}$$

where N represents the number of subjects.

### 4.1. Experiment Based on Publicly Available Datasets

The public datasets used in this paper are Ninapro DB2 and Ninapro DB4. The DB2 dataset was signalized using Trigno Wireless active dual-differential wireless electrodes, and a total of 40 sparse sEMG data were collected from 40 healthy subjects (29 males and 11 females) [35]. The dataset consisted of forty-nine gestures divided into three main categories, the first category (E1) consisted of eight uniformly open and closed gestures of equal length and nine wrist-based gestures, the second category (E2) consisted of twenty-three grasping and functional gestures, and the third category (E3) consisted of nine different finger exertion patterns. The DB4 dataset was acquired using twelve Cometa wireless electrodes, and the data were collected from ten healthy subjects (six males and four females). The dataset consisted of a total of 52 gestures divided into three main categories, the first category (E1) consisted of 12 basic finger movements, the second category (E2) consisted of 17 isometric and isotonic hand gestures and basic wrist movements, the third category (E3) consisted of 23 grasping and functional gestures. During the experimental acquisition, each gesture of the subjects was repeated six times for 5 s with a 3-s rest, and the sampling frequency was 2000 Hz, with the mean square value of the baseline noise less than 750 mV. To obtain the sEMG grayscale images, the same strategy as in previous studies [36] was followed, with the window length w fixed at 200 ms, the length of the step window s set to 50 ms, and the size of the obtained sEMG grayscale images was 400×12×1. For dataset division, the 1st, 3rd, 4th, and 6th repetitions were used as the training set, and the 2nd and 5th repetitions were used as the test set for each subject according to the number of repetitions of each action. The specific parameters of the dataset are shown in Table 2.

#### 4.1.1. Ablation Experiments

To verify the role of MS Block, ResCBAM, and Bi-LSTM proposed in this paper in the whole model, we use ablation experiments to analyze these structures layer by layer. In the model training process, Adam with adaptive learning rate adjustment characteristics is uniformly used as the optimizer, and the cross-entropy loss function, which is widely used in multi-classification problems, is used for forward propagation, and the cross-entropy loss function is shown in Equations (6) and (7).

(6) $$loss=\frac{1}{S}\sum_{i}^{S}loss_{i}$$

(7) $$loss_{i}=-\sum_{c=1}^{M}y_{ic}\log\left(p_{ic}\right)$$

where loss is the average loss rate; $loss_{i}$ is the loss rate of i samples; S is the total number of samples; M is the number of categories; $y_{ic}$ takes the value of 0 or 1, if the real category of the first sample is equal to c, then take 1, otherwise, take 0; $p_{ic}$ is the predicted probability that the observed sample belongs to c.

Five subjects (S3, S8, S15, S36, S40) were selected in DB2, and three subjects (S1, S4, S7) were selected in DB4, and their gesture data were used as the base data for the ablation experiments. The comparison architectures for the ablation experiments are M1 (RNN, consisting of three layers of Bi-LSTM), M2 (ResCBAM), M3 (ResCBAM + MS Block), and M4 (MS-CLSTM: ResCBAM + MS Block + Bi-LSTM). The results of the ablation experiments are shown in Table 3, from which it can be seen that with the gradual improvement of the model structure, the accuracy of gesture recognition is significantly improved. The specific conclusions are as follows: for the base model M1, the performance is relatively poor, with an average accuracy of 71.05% (DB2) and 67.11% (DB4) on the two datasets, indicating that the RNN alone is not sufficient to effectively extract the complex features of sEMG. The M2 model significantly improves the recognition accuracy, reaching 86.98% (DB2) and 82.57% (DB4), which indicates that the module has a significant effect on the feature extraction of sEMG. The M2 model, by integrating Simple-ResNet and CBAM, not only enhances the model’s focus on the key features but also improves the overall performance of the model by effectively capturing the correlation information between the channels. The M3 model, which incorporates the MS Block on top of ResCBAM, further improves the recognition accuracies of 87.92% (DB2) and 83.54% (DB4), which indicates that multi-scale fusion can capture the information of the input signals more comprehensively, thus improving the model’s ability to learn the complex features of the sEMG image. The M4 model, which incorporates ResCBAM, MS Block, and Bi-LSTM, achieves the best performance on both datasets with 89.50% (DB2) and 84.88% (DB4). This indicates that MS-CLSTM, which combines a convolutional neural network, attention mechanism, multi-scale feature extraction, and recurrent neural network, can better adapt to the complexity of sEMG and significantly improve the accuracy of gesture recognition.

Overall, the MS-CLSTM model proposed in this paper achieves high accuracy in sEMG gesture recognition tasks, proving its feature extraction and classification effectiveness. By introducing the CBAM and MS Block modules, the model can better capture important features in sEMG, and the combination of Bi-LSTM further improves the model’s ability to process temporal information, resulting in a significant performance improvement in the gesture recognition task. This end-to-end model, which integrates multi-scale feature extraction and timing feature capture, can understand and learn the feature representation of sEMG more deeply at different scales and provides an efficient and accurate solution for gesture recognition based on sEMG images, which is of great theoretical and applied significance.

#### 4.1.2. Gesture Recognition Experiment

**Figure 8:**
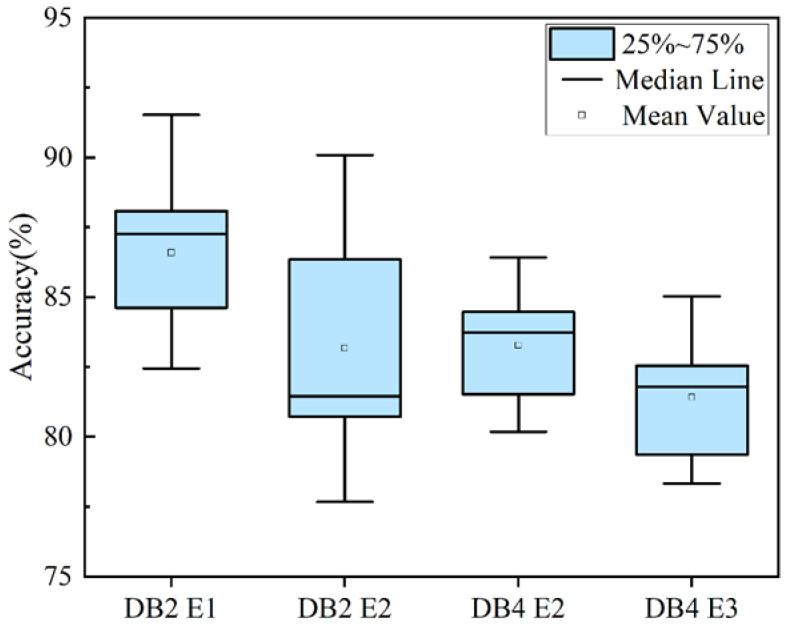
Overall accuracy box plots for the MS-CLSTM.

This section presents gesture recognition experiments using the MS-CLSTM model based on two public datasets (Ninapro DB2 and Ninapro DB4). The experiments utilized the same input size: 400 × 12 × 1. Two sub-datasets from each dataset were analyzed: E1 (17 gestures) and E2 (23 gestures) for DB2, and E2 (17 gestures) and E3 (23 gestures) for DB4. Table 4 displays Ninapro DB2 (E1, E2) and Ninapro DB4 (E2, E3), while Figure 8 shows box-and-line plots of recognition accuracy based on the two datasets. These plots illustrate the distribution of recognition accuracies for each dataset, including statistics such as quartiles, medians, and means. For Ninapro DB2, the average accuracy of the 17 gestures in E1 is 86.66%, with a recognition rate variation range of 10.08%. The box-and-line plot indicates that the median recognition accuracy is close to the mean, and the distribution is relatively balanced without any significant skewness or extremes. The box-and-line plot for the 23 gestures in E2 shows an average accuracy of 83.17% with a recognition rate variation range of 11.41%. The width of the box is greater than that of E1, and the distribution is relatively decentralized, but there are no outliers. In the case of Ninapro DB4, the 17 gestures in E2 have an average accuracy of 83.27% and a recognition rate variation range of 6.24%. The boxplot shows that the median recognition accuracy is close to the mean, the width of the box is narrower, and its distribution is relatively balanced, with no significant skewness or extremes. The 23 gestures in E3 have an average accuracy of 81.41% and a recognition rate variation range of 6.71%. The boxplot displays a slightly wider box width, a median recognition accuracy close to the mean, and a balanced distribution.

As shown in Table 4, it can be concluded from the experimental results that for the same dataset, the average accuracy of gesture recognition for different sub-datasets performs well. Although the accuracy decreases slightly with the increase in the number of gestures, the overall fluctuation is small, indicating that the model has good adaptability in different of gesture classification tasks. For different datasets, the average accuracy of the model for gesture recognition on DB2 and DB4 shows high stability. The differences in the acquisition methods of these two datasets lead to different data characteristics. Although the average accuracy of DB4 is slightly lower than that of DB2, the fluctuation of its recognition rate is small, indicating that even with the differences in the datasets, the model is still able to effectively adapt to and deal with the variations in different types of data.

Overall, the MS-CLSTM model has high accuracy in different gesture recognition tasks. The MS-CLSTM model can effectively capture and utilize the key features in the sEMG images. Although there are fluctuations in the recognition accuracies in different datasets, the recognition results demonstrate the effectiveness and reliability of the model for sEMG gesture recognition.

#### 4.1.3. Comparative Analysis of Identification Performance of Different Models

Based on the Ninapro dataset, this experiment uses the MS-CLSTM model to recognize electromyographic gestures, and the experimental results are compared with the mainstream recognition methods of other research scholars, as shown in Table 5. The results show that compared with other recognition methods, the MS-CLSTM model outperforms most existing methods on both datasets, and MS-CLSTM integrates the use of multi-scale feature extraction, a residual network, attention mechanism, and a bidirectional long short-term memory network, which can more accurately capture the details and overall features of the sEMG, thus effectively improving the accuracy and stability of gesture recognition. This result shows that the MS-CLSTM model has certain advantages in feature extraction and recognition ability based on sEMG.

### 4.2. Real-Time Performance Metrics and Results

In this section, the proposed MS-CLSTM model is used for the gesture recognition task in the real-time control system of a myoelectric manipulator. First, the sEMG corresponding to different gestures is collected from the experimenter, and then the MS-CLSTM model is trained to obtain the optimal performance sEMG gesture recognition model. After the model training is completed, the optimal MS-CLSTM gesture recognition model is integrated into the real-time control system of the myoelectric manipulator. The system processes the sEMG data efficiently and converts them into accurate gesture recognition results, providing reliable support for the real-time control of the manipulator.

**Figure 9:**
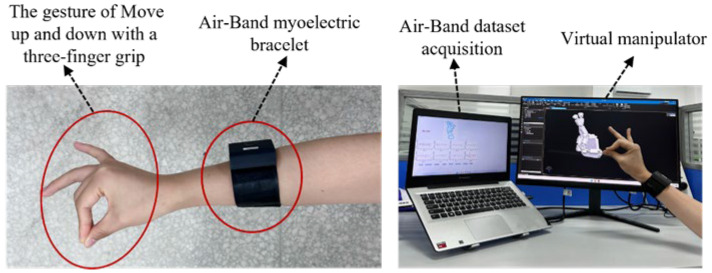
Experimental scene diagram.

**Figure 10:**
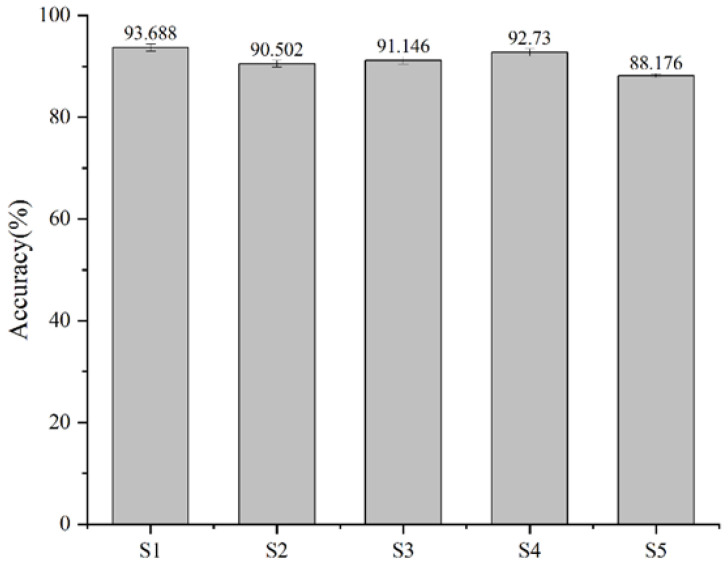
The recognition rate of 5 subjects based on MS-CLSTM.

We collected sEMG data from five experimenters, four males and one female. The experimenters wore Air-Band bracelets, and six gestures were acquired for each experimenter, as shown in Figure 9. Each gesture of each subject was acquired for five repetitions, the sampling frequency was 1000 Hz, the number of acquisition channels was eight, and each subject had 30 actions in each round of data acquisition (a cycle of six actions with five repetitions of each action, each action lasting for 5 s, after each data acquisition cycle, subjects rested for 1 min before proceeding to the next cycle). To obtain a sample sEMG grayscale image, the window length w in Equation (2) was fixed to 100 ms, the length of the step window s was set to 50 ms, and the image input size during the experiment was 200×8×1. Next, the MS-CLSTM model was trained, and the 1st, 2nd, 4th, and 5th repetitions were used as the training set, and the 3rd repetition was used as the test set. Five cross-validations were performed on five experimenters (S1 to S5), and the results of the experiment are shown in Figure 10, which is a bar chart demonstrating the average of the five cross-validations for each subject with error bars. The average gesture recognition accuracy for the five subjects is around 90%, indicating that the model performs consistently and well on different individuals. S1 and S4 have the highest accuracy rates, 93.68% and 92.73%, both reaching over 90%. The accuracy of S5 is relatively low but can still reach over 85%. The error bar of S4 is slightly longer, which indicates that the test results of this subject fluctuate in some cases. The longer error bar of S4 may be due to individual differences or some inconsistent factors in the data collection process. The shorter length of the error bars for all subjects except S4 indicates that the model performed consistently on most subjects. In general, these errors did not affect the overall high accuracy. The accuracy rate of each subject is close to or over 90%, which verifies the general applicability and validity of the model on different individuals and lays the foundation for realizing the real-time control system of the manipulator.

After the model training is completed, the optimal MS-CLSTM myoelectric manipulator gesture recognition model is integrated into the myoelectric manipulator real-time control system. The control system contains a signal acquisition module, a signal processing and gesture recognition module, and an actuator, and the overall implementation of the real-time control system of the myoelectric manipulator is shown in Figure 9. The signal acquisition module collects the sEMG of the arm in real time through the wireless eight-channel sEMG armband, and the signals are then transmitted to the computer in real time by Bluetooth. The virtual manipulator of the actuator is established by SolidWorks (2022) and Siemens NX 1980 animation software and is directly driven by the control commands generated by the gesture recognition module to complete the actions. The virtual manipulator defines the motion of 16 joints, configures the motion vice and joint drive, and sets up a total of six action modes: (1) index finger action, (2) three-finger grip up and down movement, (3) thumb left and right sliding, (4) vertical thumb, (5) two-finger grabbing release, (6) fist clenching. Each action corresponds to a different hand mode, and the system accomplishes different hand grasping modes by adjusting the rotation angle of each joint of the manipulator.

For practical application scenarios (e.g., human–computer interaction, robotics, and assistive technologies), it is critical that the myoelectric manipulator gesture recognition system can accurately recognize real-time gestures, thus the real-time nature of the gesture recognition model determines the usability of the real-time control system of the manipulator. To enable the user to control the myoelectric manipulator in real time, fast response time and high prediction speed of the myoelectric manipulator gestures in the system are crucial [38], thus the real-time performance of the myoelectric manipulator grasping pattern decoding can be evaluated by prediction speed and response time.

For the real-time recognition and control of gestures for S1, Table 6 shows that the average response time of the myoelectric robotic gesture recognition model for S1 is 0.060 ± 0.005 s, which is relatively short, indicating that the model can process the input data quickly, especially in real-time applications, where response times of less than 100 ms are usually desirable. The response time fluctuations are also relatively small (±5 ms), indicating that the model is stable. The prediction speed is 15 ± 3 FPS, with 15 FPS being a moderate speed for gesture recognition tasks. The fluctuation range of the prediction speed (±3 FPS) indicates that the processing speed may vary slightly in different situations, but generally stays between 12 and 18 FPS. The response time and the fluctuation range of the prediction speed of the MS-CLSTM model are within reasonable limits, which indicates that the model is stable in different environments.

**Figure 11:**
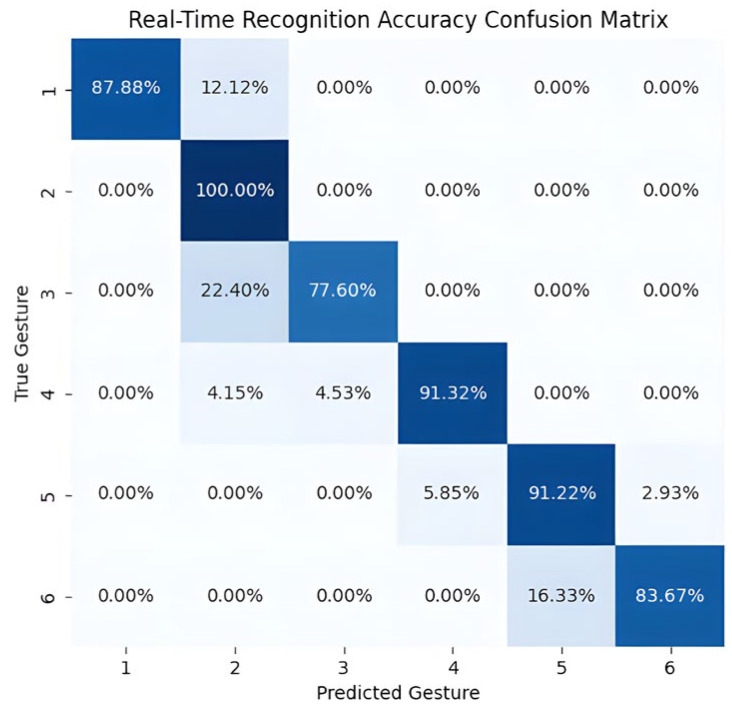
Real-Time Recognition Accuracy Confusion Matrix. (Darker blue indicates higher accuracy, with a larger number of correctly classified instances. Lighter blue indicates lower accuracy, with fewer correctly classified instances.)

Analysis based on average response time and prediction speed shows that a real-time control system for manipulators produces a large number of classification results per second. However, since the accuracy of the classifier cannot reach 100%, a certain percentage of classification errors will inevitably occur in these results. To improve the accuracy and stability of real-time recognition, this study introduces the majority voting window technique. Figure 11 shows the real-time gesture recognition accuracy confusion matrix for experimenter S1 under a voting window of size 100. Overall, the average recognition accuracy of the six gestures is 89%. Among them, the accuracies of gesture 2 (moving up and down with a three-finger grip) and gesture 4 (giving a thumbs up) are 100% and 91.32%, respectively. This indicates that the sEMG features of these two gestures are clearly distinguishable and can be reliably recognized by the model. The recognition accuracy of gesture 1 (index finger movement) was 87.88%, but 12.12% of the samples were misclassified as gesture 2 (three-finger grip moving up and down), and this confusion may be due to the similarity of the signal features of gesture 1 and gesture 2 at certain periods, which made it difficult for the model to accurately distinguish them. In addition, there is a certain degree of confusion between gesture 3 (thumb sliding left and right) and gesture 4 (thumbs up), the recognition accuracy of gesture 3 is 77.60%, but 22.40% of the samples are misclassified as gesture 4. This phenomenon suggests that the sEMG data of gesture 3 are closer to the signal characteristics of gesture 4 in some specific windows, which leads to misclassification by the classifier. The recognition accuracy of gesture 5 (two-finger grasping and releasing) was 91.22%, but 5.85% of the samples were misclassified as gesture 4, while gesture 6 (clenched fist) had a recognition rate of 83.67%, of which 16.33% of the samples were misclassified as gesture 5. The confusion between gesture 5 and gesture 6 may be because the two gestures have more similar signal characteristics under some specific conditions, especially after the windowing process, and certain subtle feature differences are further weakened, leading to a decrease in the model’s discriminative ability. To summarize, the recognition performance of gesture 2 and gesture 4 is more satisfactory, while the signal similarity between gesture 1 and gesture 2, gesture 3 and gesture 4, and gesture 5 and gesture 6 leads to a certain degree of confusion.

Overall, during the process of real-time recognition and control, the recognition accuracy of individual gestures fluctuated due to the feature similarity between gestures and other reasons, but the overall performance was still satisfactory. This indicates that the MS-CLSTM model has strong ability in the sEMG gesture recognition task, and the recognition model has stable results for three-finger grip up and down movement and thumbs up.

## 5. Discussion

The MS-CLSTM model proposed in this study integrates multi-scale feature extraction, ResCBAM, and Bi-LSTM, effectively enhancing the accuracy and robustness of gesture recognition from sEMG signals. The results from the Ninapro DB2 and DB4 datasets, as well as our own Air-Band dataset, consistently demonstrate that the MS-CLSTM outperforms existing gesture recognition models in terms of recognition accuracy and generalizability. Furthermore, the model’s real-time performance, with a response time of approximately 0.060 s and a prediction speed of 15 frames per second (FPS), indicates its suitability for integration into real-time control systems.

From the perspective of previous studies, this research aligns with the advancements made in using deep learning models for sEMG-based gesture recognition. Studies like those of Geng et al. and Wei et al. have employed convolutional neural networks (CNNs) and hybrid CNN-LSTM architectures to extract both spatial and temporal features of sEMG. However, our model introduces a novel approach by leveraging multi-scale convolutions to capture both local and global patterns within the sEMG data, while also integrating the ResCBAM module to emphasize salient signal features. This combination addresses limitations in earlier works that relied solely on single-scale convolutions or standard attention mechanisms.

Although the MS-CLSTM model demonstrates significant advancements in accuracy and real-time performance, it still has certain limitations. While the model performs well on the current datasets, its generalization ability on larger and more diverse datasets has yet to be fully validated. In practical applications, factors such as sensor placement, individual differences, skin conditions, and muscle fatigue may affect the model’s robustness. Future work could address these issues by optimizing the model and training it on larger datasets, thereby promoting its broader application in prosthetics, robotic control, and other human–computer interaction scenarios.

## 6. Conclusions

In this paper, a novel MS-CLSTM sEMG gesture recognition model is proposed by integrating a multi-scale feature extraction module, residual convolutional block attention mechanism (ResCBAM), and bidirectional long short-term memory network (Bi-LSTM). The model effectively captures the local details, global contextual information, and temporal variations of sEMG, resulting in high recognition accuracy and stability in complex gesture recognition tasks. Our proposed MS-CLSTM model shows significant improvement in accuracy compared to classical machine learning methods as well as the current mainstream deep learning models CNN and CNN-LSTM. In the myoelectric manipulator gesture control task, the real-time recognition accuracy reaches 89%. Overall, the MS-CLSTM model demonstrates significant advancements in accuracy and real-time performance. Future work could optimize the model for larger datasets, reduce computational demands, and achieve low-power deployment, facilitating broader applications in prosthetics, robotic control, and other human-computer interaction scenarios.

## Figures and Tables

**Table 1:** Parameter Settings of Multi-Scale Block.

Multi-Scale Block	Small Scale	Big Scale
Convolution kernel size (Con2D)	5 × 1	10 × 1
	8 × 1	20 × 1
	1 × 2	1 × 4
Maxpooling size	2 × 2	2 × 2
Number of convolution kernels	8	8

**Table 2:** Details of Two publicly available sEMG datasets.

Details	Ninapro DB2	Ninapro DB4
Number of classified gestures	49	52
Intact subjects	40	10
Electrodes	12 Delsys	12 Cometa
Sampling rate (Hz)	2000	2000
Number of trials	6	6

**Table 3:** The classification and performance comparison of Ablation studies.

Subject		Accuracy (%)			
		M1: RNN	M2: ResCBAM	M3: ResCBAM + MS Block	M4: MS-CLSTM
DB2	S3	78.55	88.33	89.45	91.52
	S8	69.45	87.23	88.05	89.88
	S15	65.03	85.42	86.47	87.26
	S36	63.28	85.72	86.35	87.91
	S40	78.94	88.19	89.27	90.95
	Average	71.05	86.98	87.92	89.50
DB4	S1	70.17	81.56	82.68	83.74
	S4	69.13	84.39	85.26	86.42
	S7	62.02	81.78	82.69	84.47
	Average	67.11	82.57	83.54	84.88

**Table 4:** The Performance Comparison of Different Data.

Dataset		Number of Movements	Average Accuracy (%)	Rate Distribution (%)
DB2	E1	17	86.66	10.08
	E2	23	83.17	11.41
DB4	E2	17	83.27	6.24
	E3	23	81.41	6.71

**Table 5:** Comparison of the classification performance by the proposed network and other methods.

Reference	Dataset	Methods	Average Accuracy (%)
Geng [19]	DB2	ConvNet	77.8
Wei [20]	DB2	Multi-view	85.8
	DB4	Pooling Network	72.9
Wei [21]	DB2	Multi-view CNN	83.7
Kim [23]	DB2	CNN-LSTM	83.91
Hu [24]	DB2	CNN-RNN	82.2
Wang [25]	DB2	TDCT	83.55
Shen [37]	DB2	CviT	83.47
Our Work	DB2	MS-CLSTM	86.66
	DB4		83.27

**Table 6:** The real-time performance of MS-CLSTM.

Model	Average Response Time (s)	Prediction Speed (fps)
MS-CLSTM	0.0600 ± 0.005	15 ± 3

## Data Availability

Our study uses anonymized sEMG for prosthetic hand control. The data are non-invasive, pose no risk to participants, and do not contain sensitive personal information or commercial interests. Therefore, the study qualifies for exemption from Ethics Committee review under the Measures for Ethical Review of Life Science and Medical Research Involving Humans (Article 32).
